# Supplementary figures and images for: TGF-β1 activates neutrophil signaling and gene expression but not migration
Source: PLoS One. 2023 Sep 8;18(9):e0290886. doi: 10.1371/journal.pone.0290886 (PMC10490904; doi:10.1371/journal.pone.0290886)

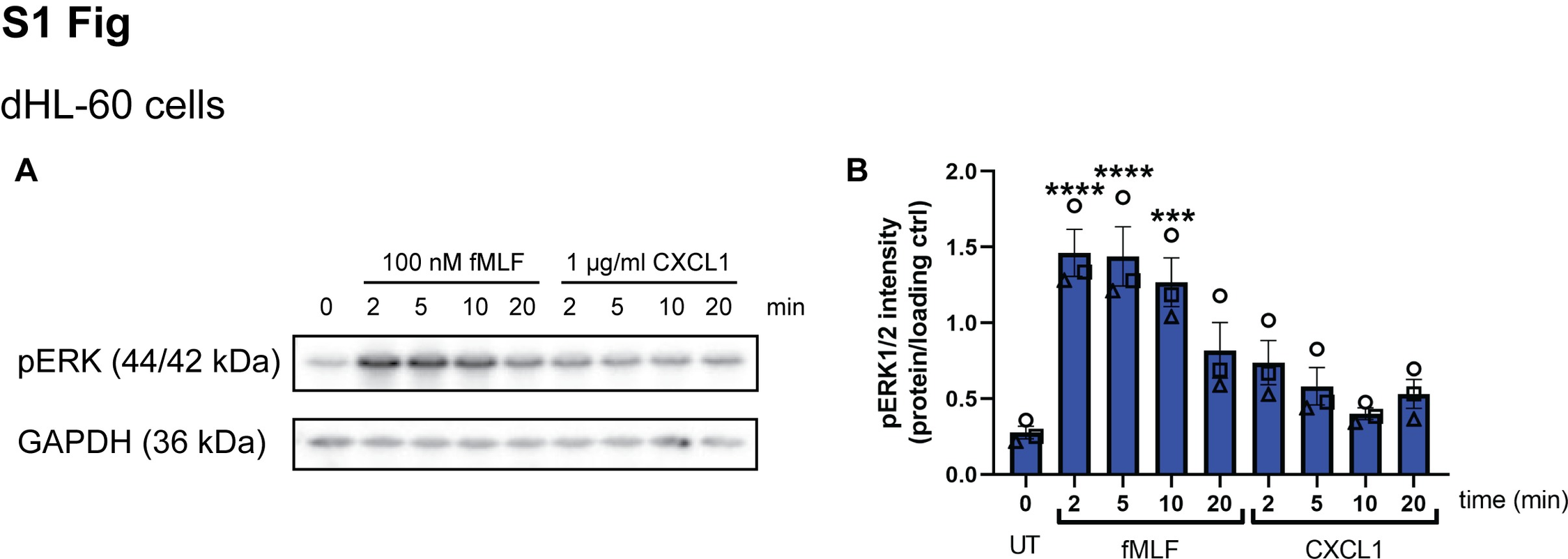

Supplement: S1 Fig — (A) Immunoblots of pERK1/2 and GAPDH in dHL-60 cells in response to fMLF or CXCL1 from 0 to 20 min. (B) Quantification of (A). ***P ≤ 0.0001, ****P ≤ 0.0001 when compared with time 0 (untreated (UT)) (one-way ANOVA with Dunnett’s multiple comparisons test). Raw data are available in S1 and S2 Files. (TIF) [file pone.0290886.s001.tif]

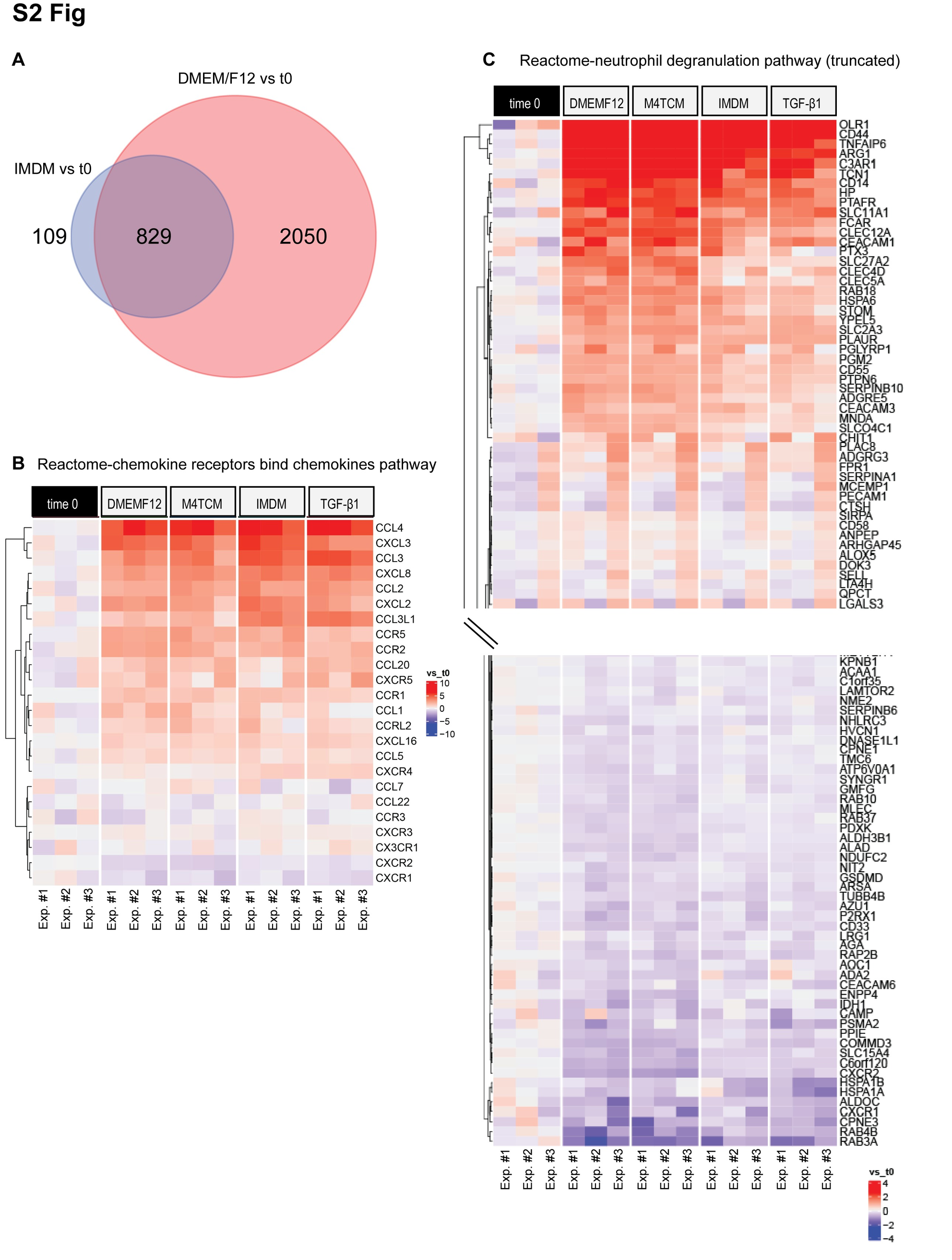

Supplement: S2 Fig — (A) Venn diagram depicting the number of changed genes in the IMDM and DMEM/F12 media conditions when each were compared to untreated control (t0). (B, C) Heat maps of Log2 Fold Change to mean time 0 expression in the (B) ‘chemokine receptors bind chemokines (R-HSA-380108)’ reactome pathway and the (C) ‘neutrophil degranulation (R-HSA-6798695)’ reactome pathway in dHL-60 cells either untreated (time 0) or treated with media controls (IMDM or DMEM/F12), TGF-β1, or M4 TCM for 30 min. Heat maps in (C) depict the 30 pathway genes with the largest average upregulation or the 30 genes with the largest downregulation relative to untreated. (TIF) [file pone.0290886.s002.tif]
