# Supplementary material for: TGF-β1 activates neutrophil signaling and gene expression but not migration
Source: PLoS One. 2023 Sep 8;18(9):e0290886. doi: 10.1371/journal.pone.0290886 (PMC10490904; doi:10.1371/journal.pone.0290886)
Supplement: S1 Table — (DOCX) [file pone.0290886.s003.docx]

**S1 Table:**  Significantly changed genes within specific reactome pathways.

| **Reactome Pathway** | **Gene** | **Log2FC** |
| --- | --- | --- |
| Signaling by TGF-β family members | JUNB | 1.095 |
|  | TGIF1 | 0.882 |
|  | SMAD7 | 3.375 |
|  | PAI1 | 1.724 |
| Class A1 (rhodopsin-like receptors) | ADRB1 | -1.645 |
|  | CXCL3 | -1.705 |
|  | EDN1 | -2.191 |
|  | GPR35 | 0.989 |
|  | S1PR2 | 3.137 |
|  | GPR65 | -0.619 |
|  | PTGER4 | -0.728 |
|  | S1PR1 | -2.048 |
| Signaling by interleukins | VEGFA | 1.185 |
|  | OSM | 4.279 |
|  | JUNB | 1.095 |
|  | CEBPD | -0.617 |
|  | CISH | 1.036 |
|  | SOCS1 | 1.464 |
|  | NFKBIA | 0.822 |
|  | CDKN1A | 1.592 |
|  | CLCF1 | 0.791 |
|  | DUSP6 | -0.722 |
|  | S1PR1 | -2.048 |
|  | PTGS2 | -1.380 |
