# Supplementary material for: TGF-β1 activates neutrophil signaling and gene expression but not migration
Source: PLoS One. 2023 Sep 8;18(9):e0290886. doi: 10.1371/journal.pone.0290886 (PMC10490904; doi:10.1371/journal.pone.0290886)
Supplement: S2 Table — (DOCX) [file pone.0290886.s004.docx]

**S2 Table:**  Number of genes significantly changed in each treatment when compared to either the untreated sample or the time-matched, media control.

| **Treatment** | **Compared to:** | **Number of Genes Changed** |
| --- | --- | --- |
| IMDM | untreated | 938 |
| TGF-β1 | untreated | 1085 |
|  | IMDM | 117 |
| DMEM/F12 | untreated | 2879 |
| M4 TCM | untreated | 2740 |
|  | DMEM/F12 | 27 |
