# Supplementary material for: TGF-β1 activates neutrophil signaling and gene expression but not migration
Source: PLoS One. 2023 Sep 8;18(9):e0290886. doi: 10.1371/journal.pone.0290886 (PMC10490904; doi:10.1371/journal.pone.0290886)
Supplement: S1 File — (PDF) [file pone.0290886.s005.pdf]

All images were captured using the chemiluminescent channel on an Azure 600. Marker images were captured using the “marker capture” feature. The marker for each image was cropped and overlaid onto the original chemiluminescent image using the ROI feature in ImageJ for exact placement. No other adjustments were made to the images. The first set of blots shown for each figure panel is the one shown in the main figure unless otherwise noted.

Figure 2A-B

dHL-60 cells

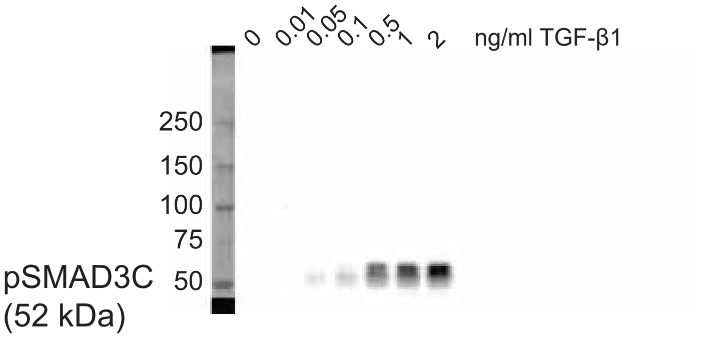

This blot was cut between 37 and 50 kD.

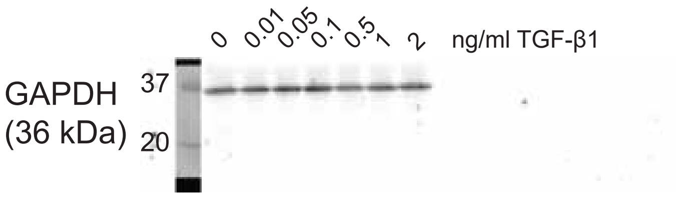

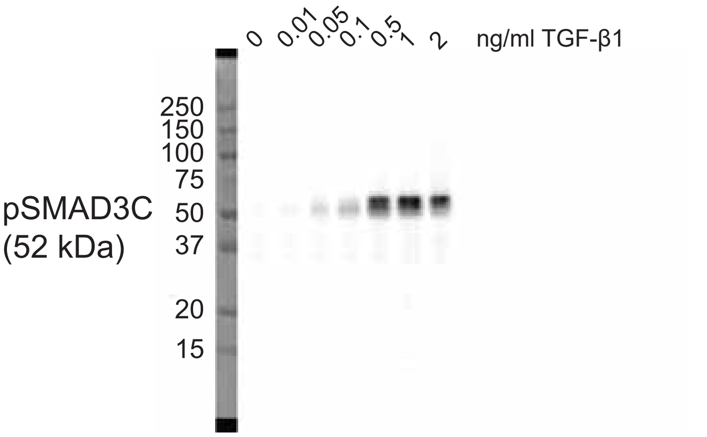

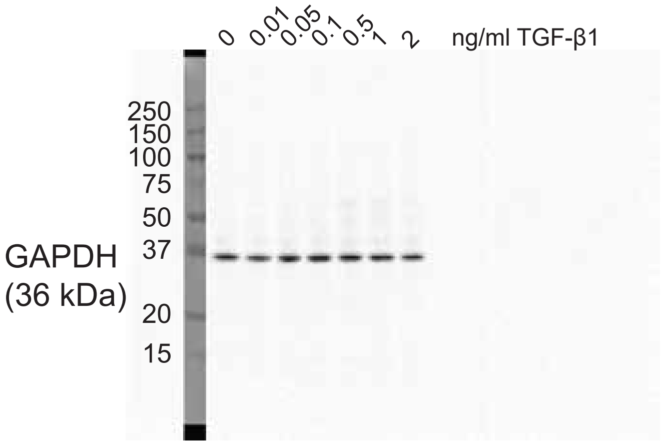

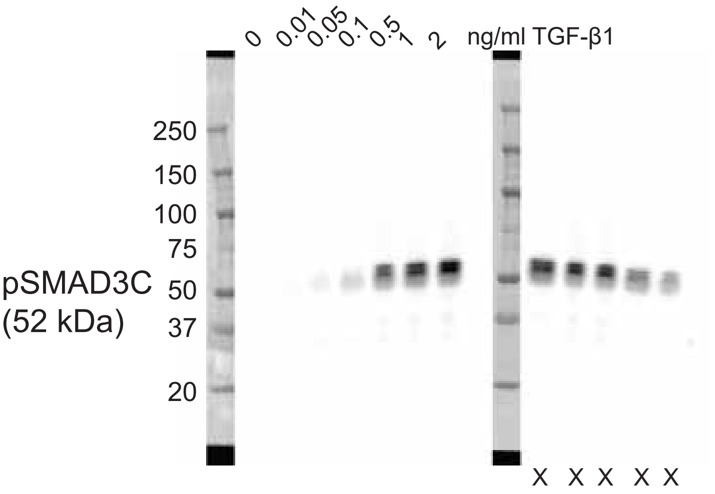

This half of the blot is samples for Figure 2G-2H.

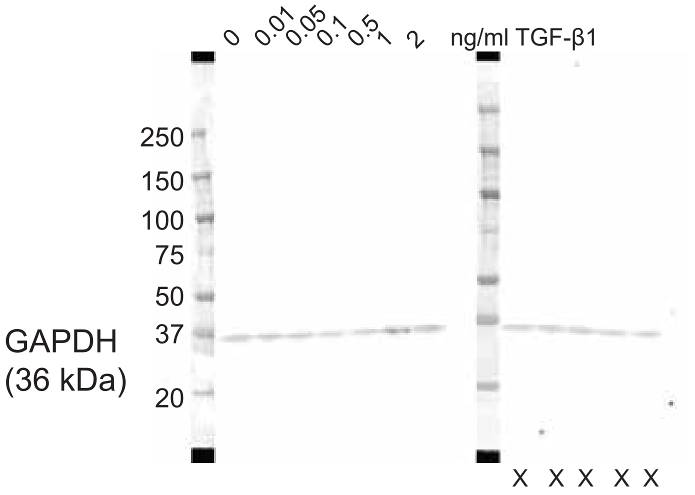

This half of the blot is samples for Figure 2G-2H.

X: Lane is not included in the final image or analysis.

Figure 2C-D

dHL-60 cells

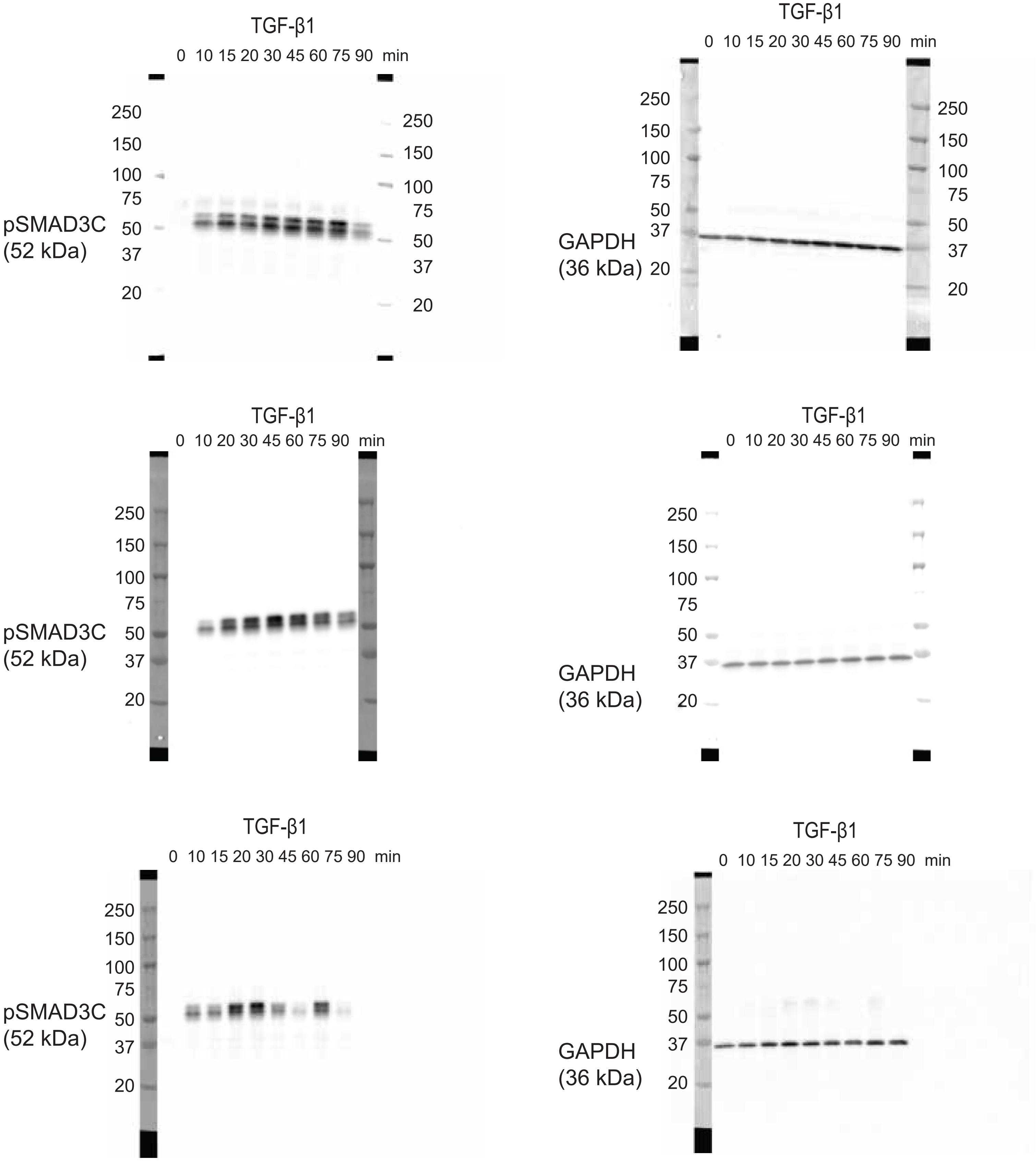

Figure 2E-F

PMNs

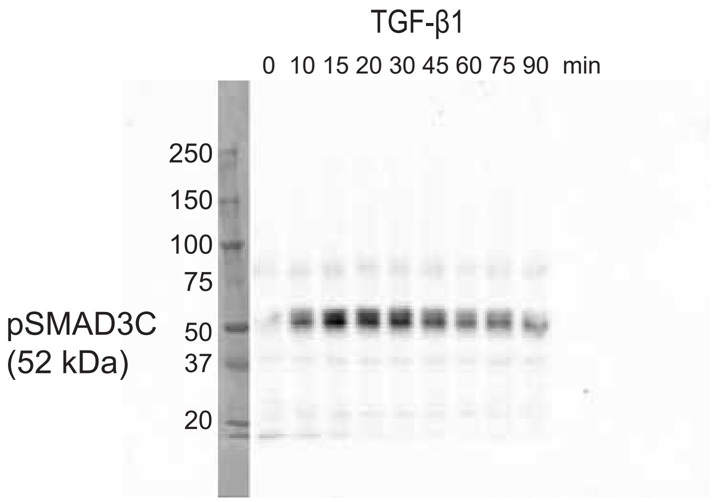

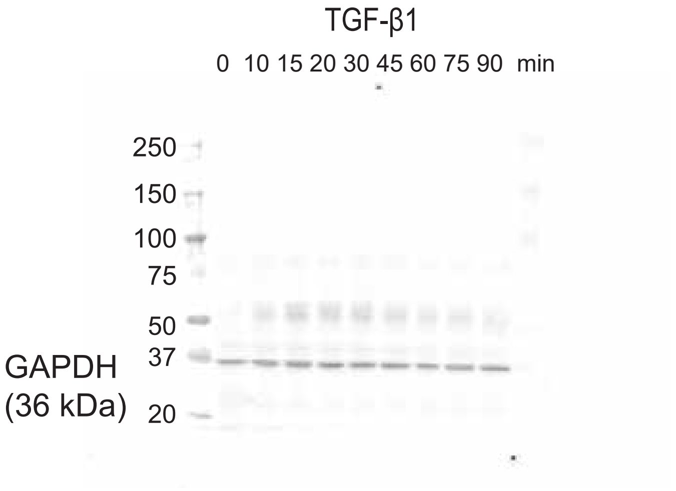

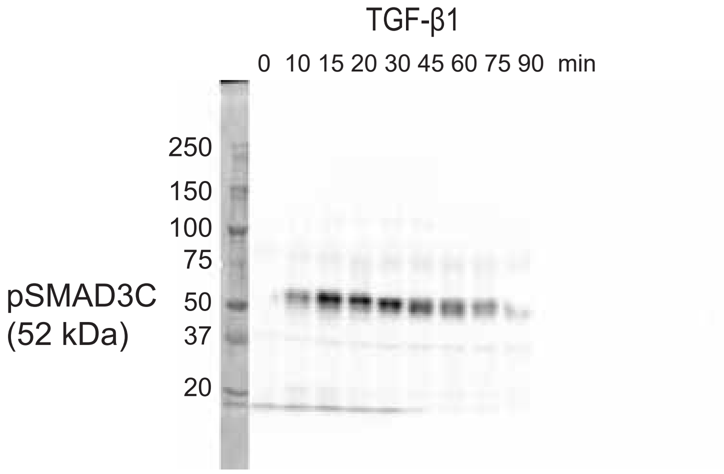

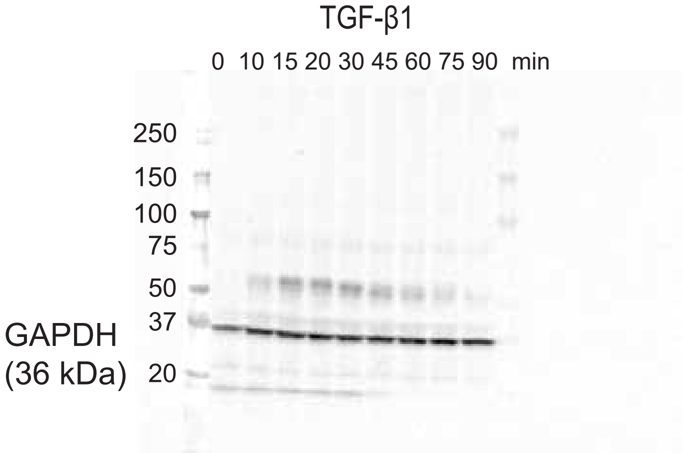

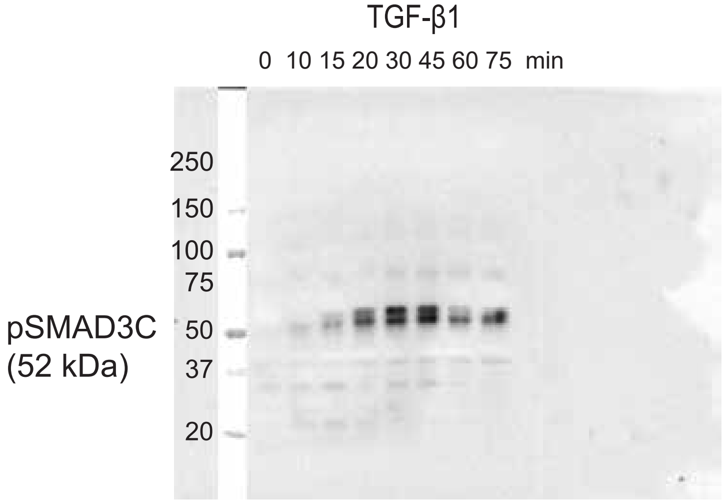

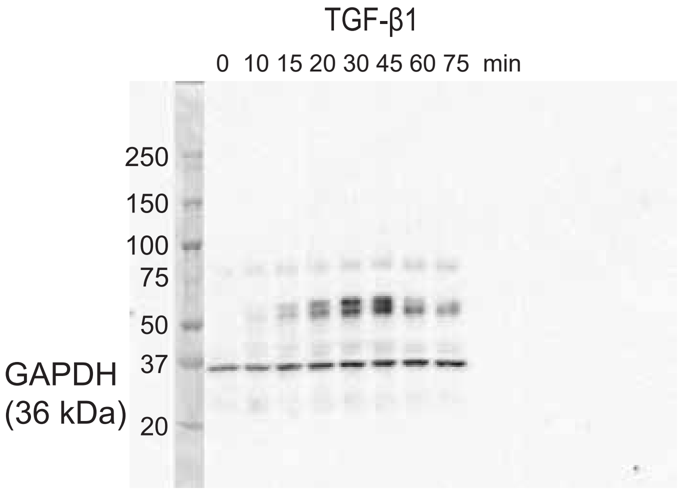

Residual pSMAD3C is seen on these loading controls.

Figure 2G-H

dHL-60 cells

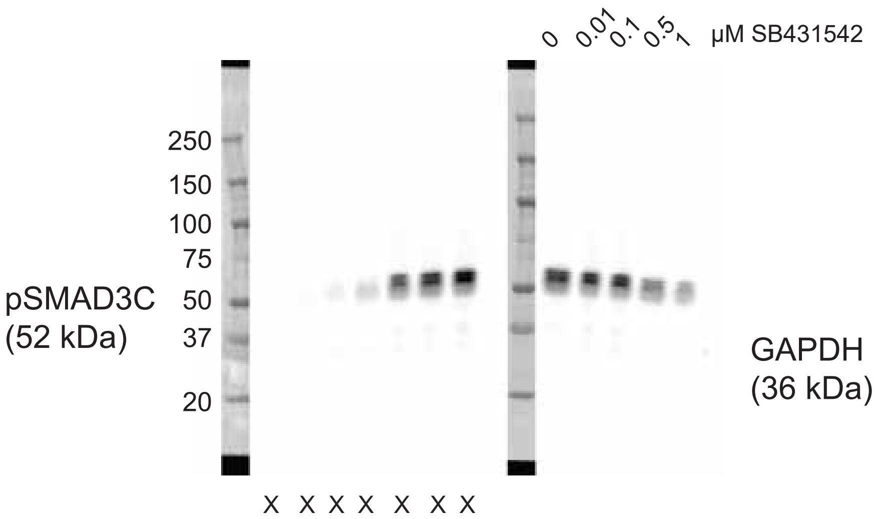

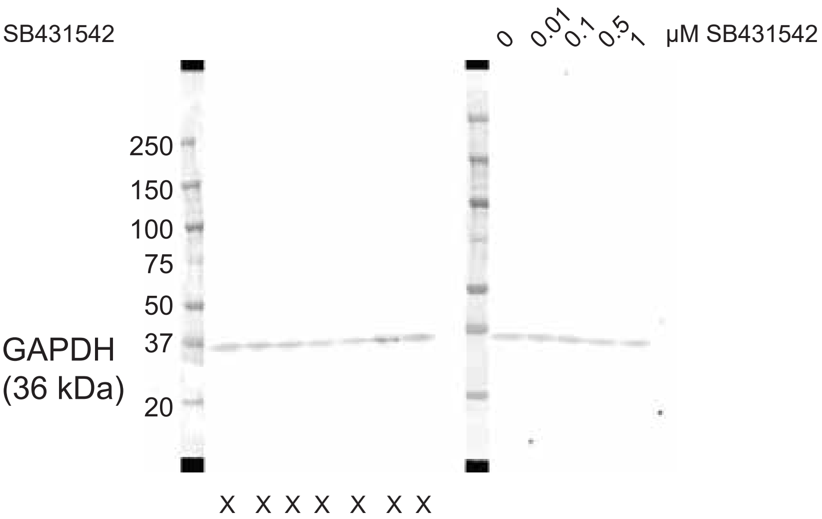

This half of the blot is samples for Figure 2A-2B.

This half of the blot is samples for Figure 2A-2B.

X: Lane is not included in the final image or analysis.

Two biological replicates were run on this gel.
This blot was cut between 37 and 50 kD.

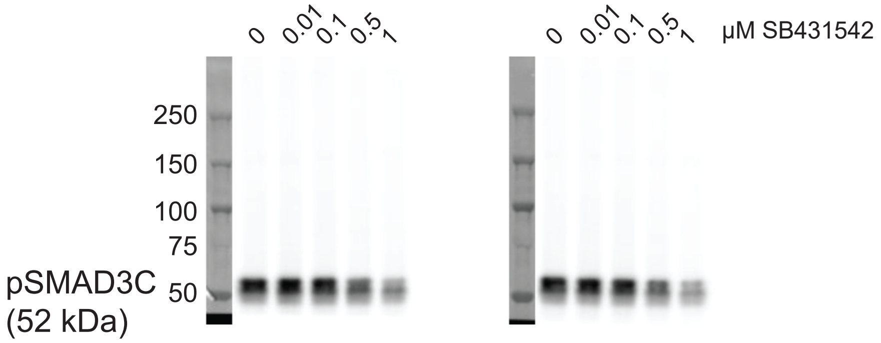

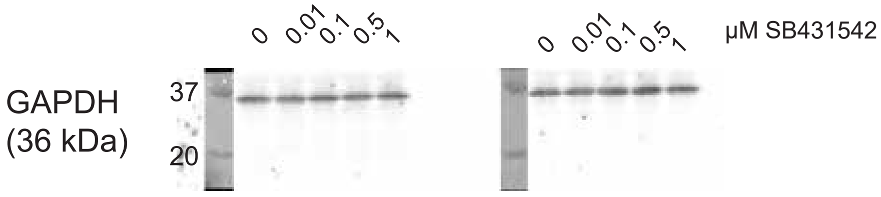

Figure 3A-B

PMNs

PMNs often show some protein degradation, even in the presence of protease inhibitors.

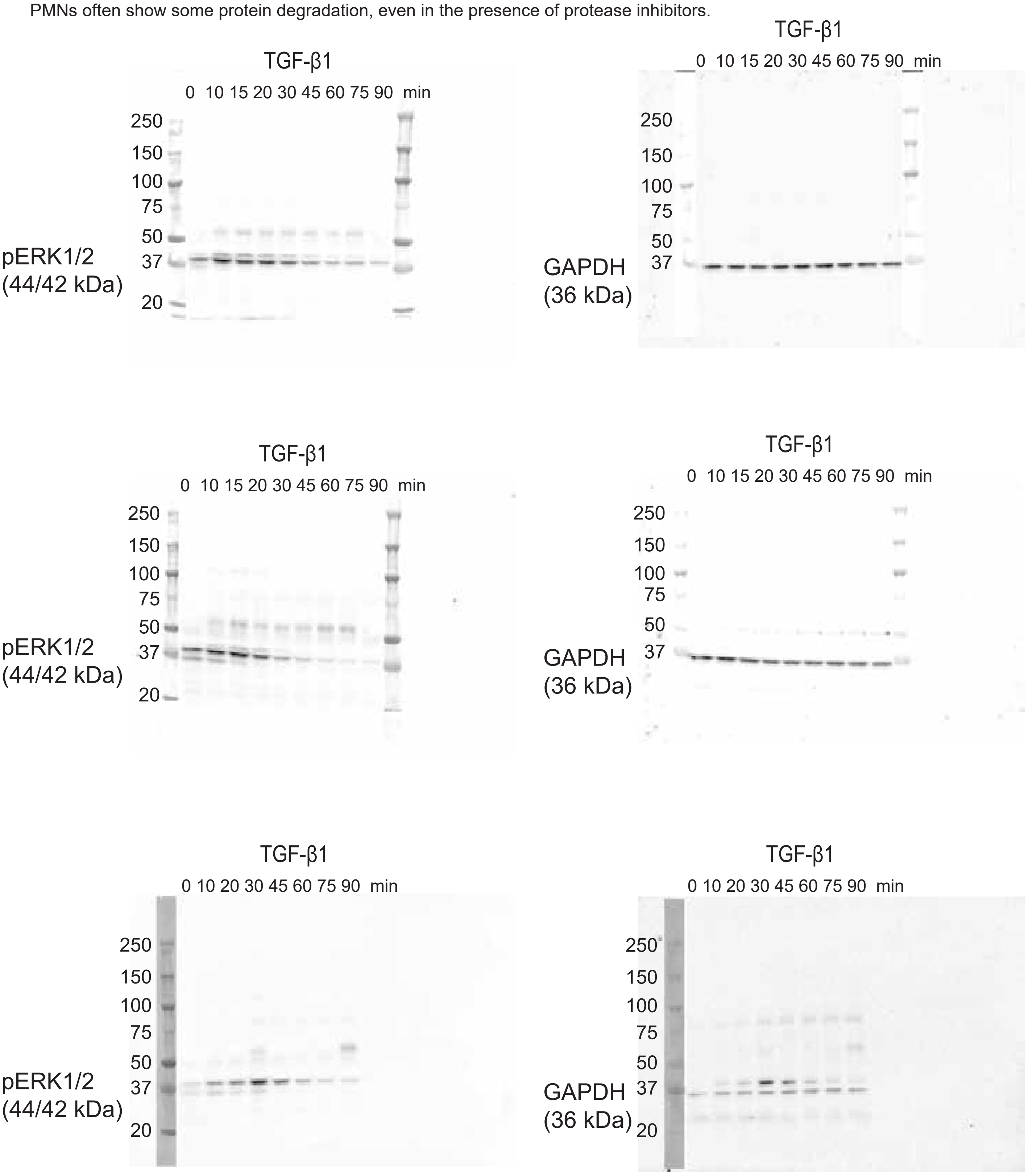

Residual pERK is seen on this image of the loading control.

Figure 3C-D

dHL-60 cells

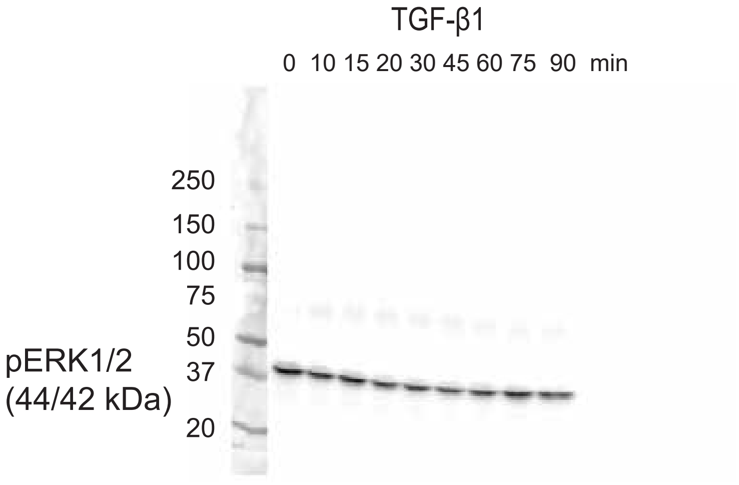

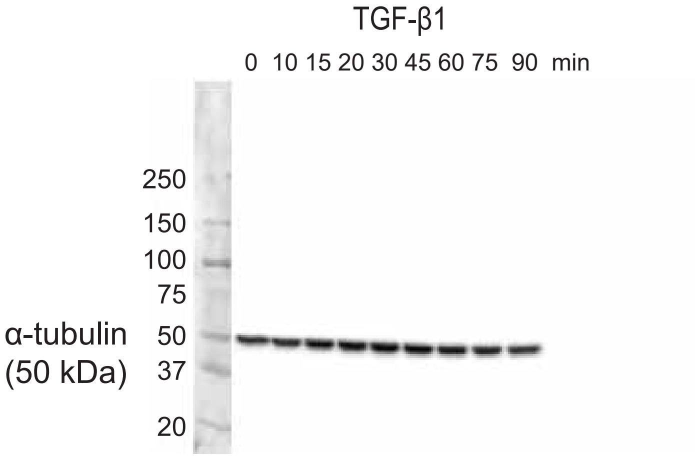

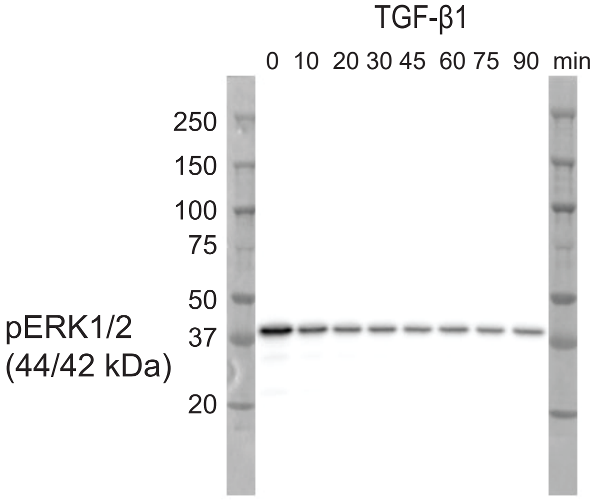

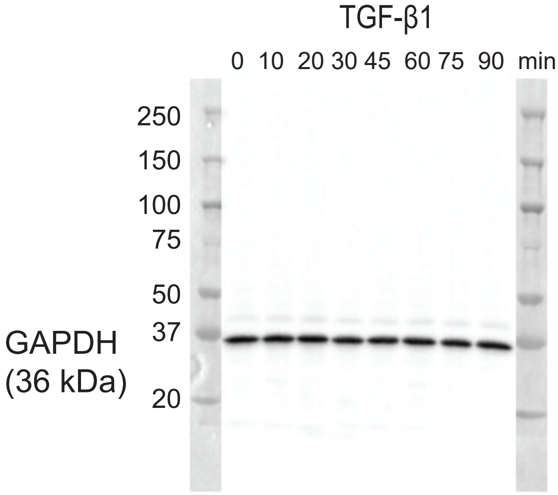

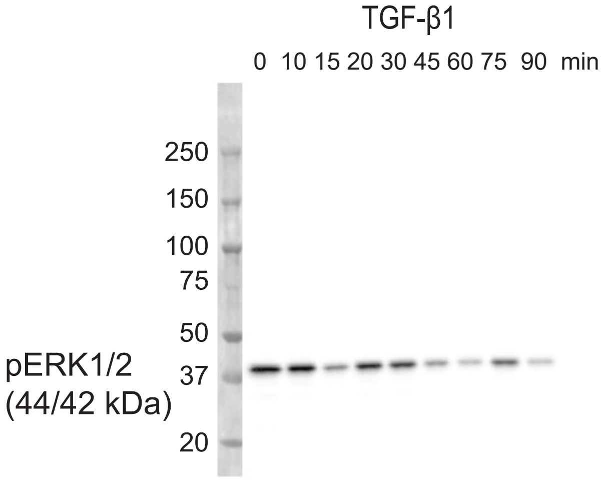

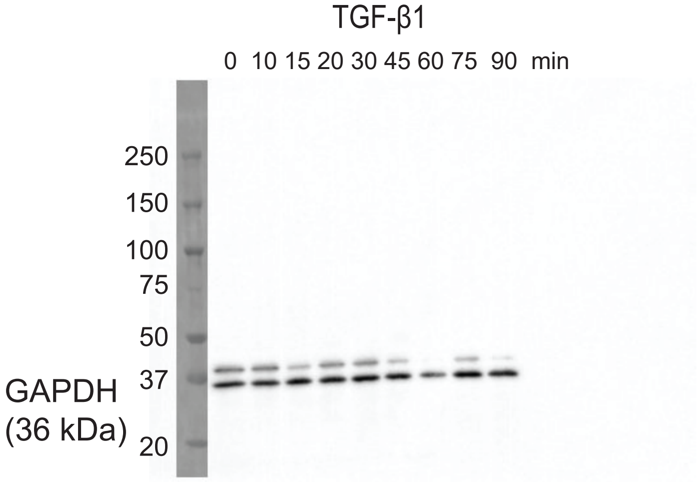

Residual pERK is seen on this image of the loading control.

Figure 4A-B

dHL-60 cells

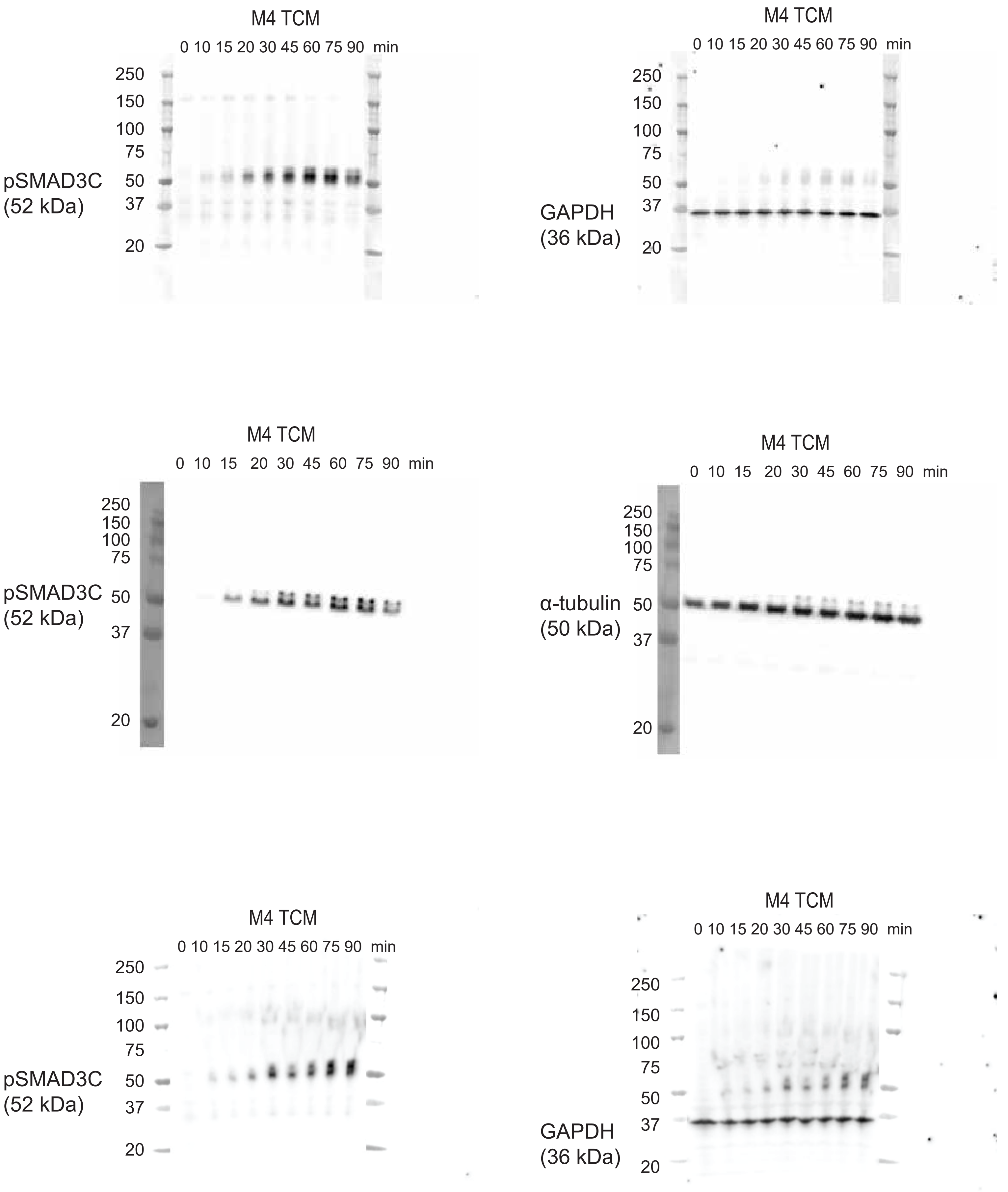

Residual pSMAD3C is seen on this image of the loading control.

Figure 4C-D

PMNs

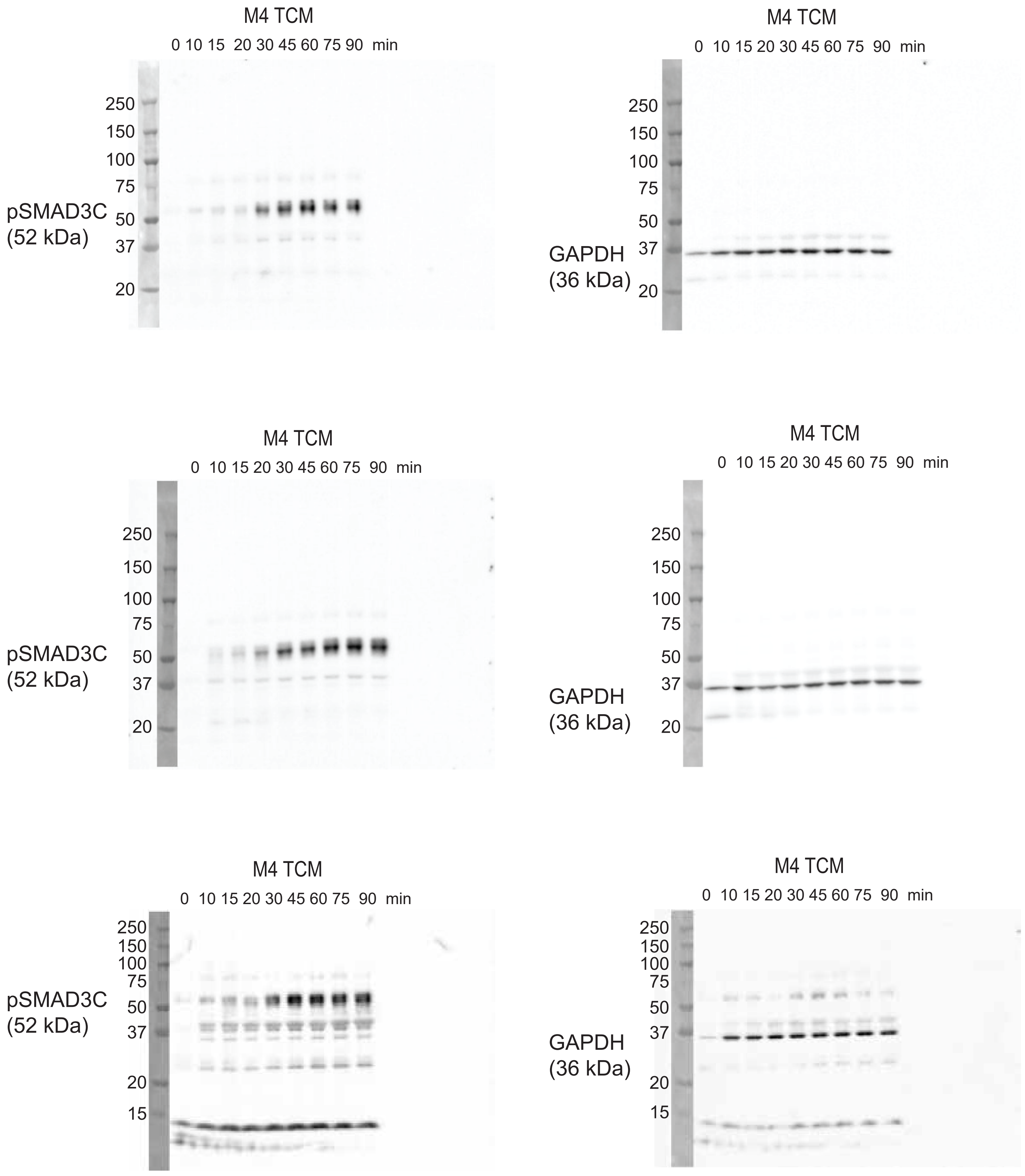

Figure 4E-F

dHL-60 cells

Both of these blots were cut between 37 and 50 kD.

Two biological replicates were run on this gel.
The right-hand set is the one used in the main figure.

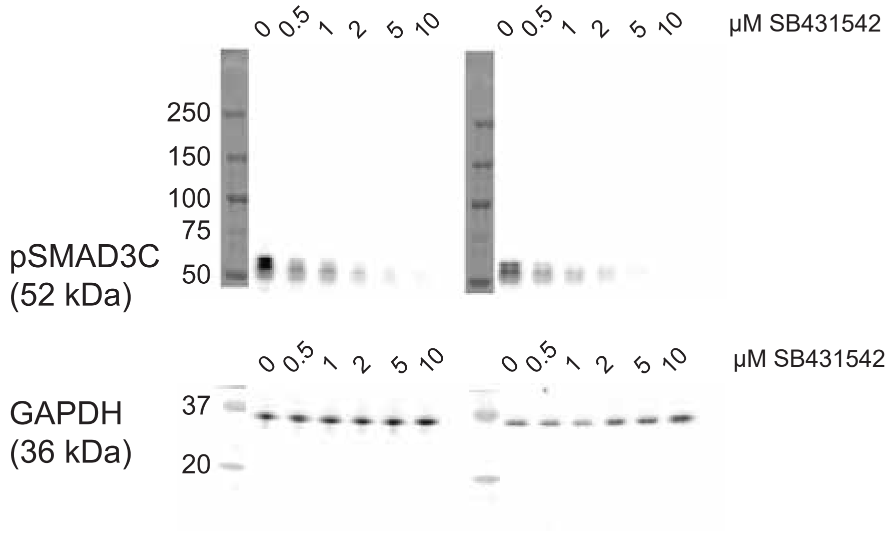

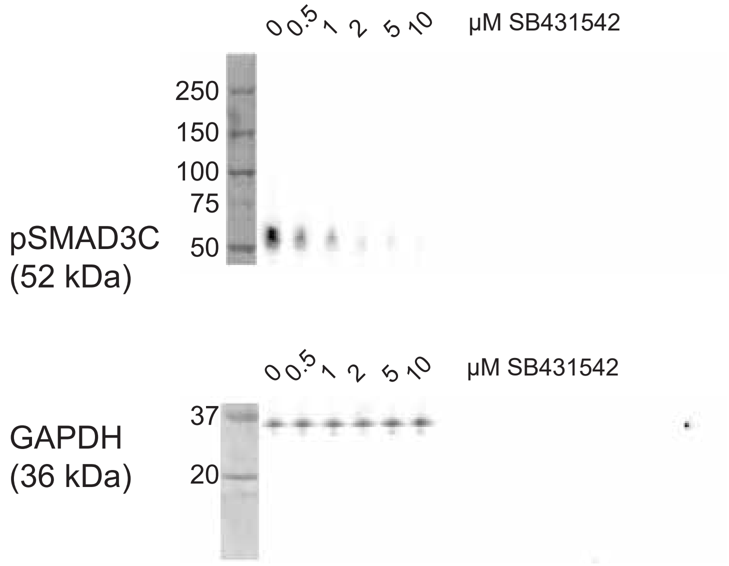

Figure 4G-H

PMNs

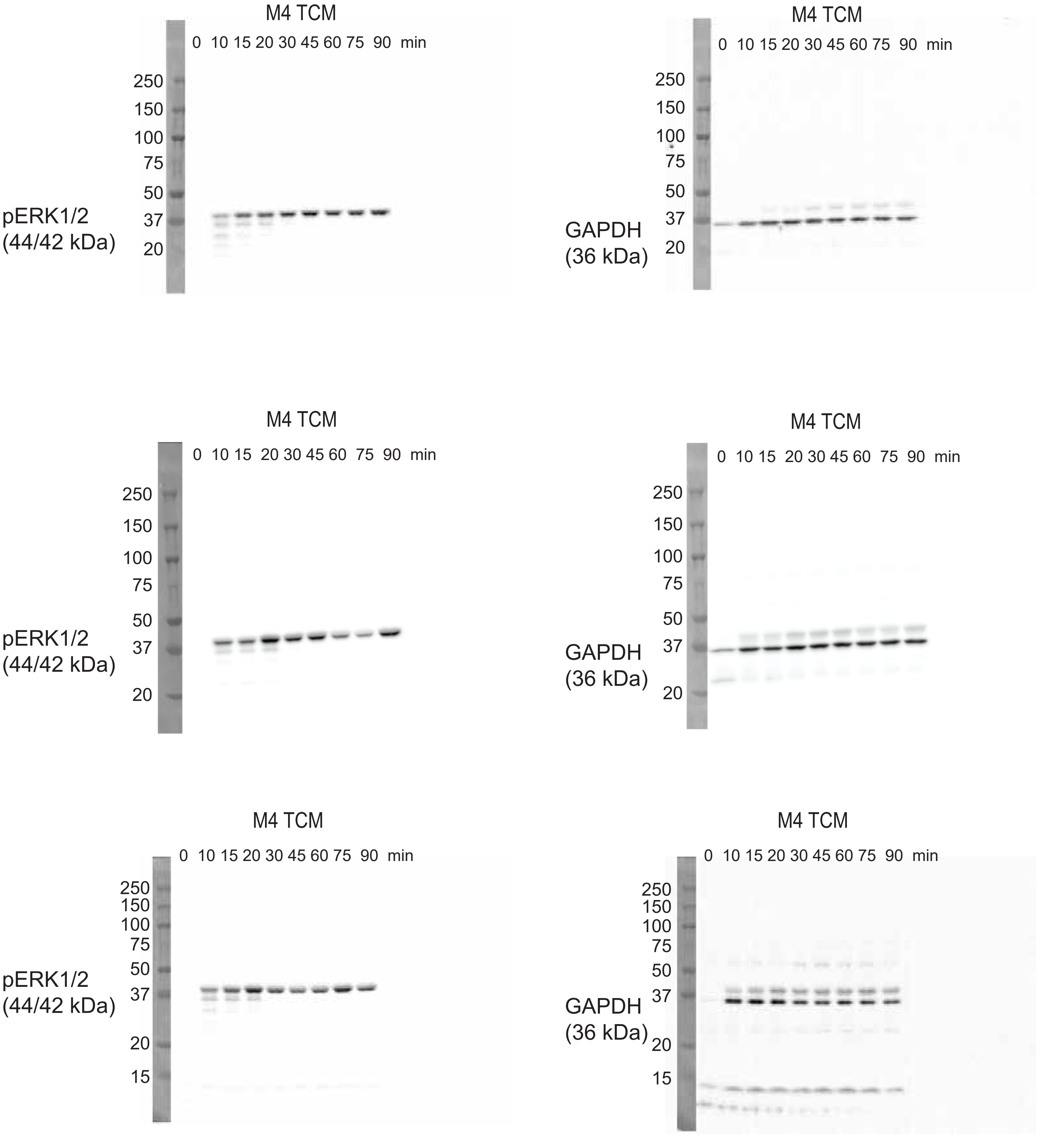

Residual pERK1/2 is seen on these loading controls.

Figure 4I-J

dHL-60 cells

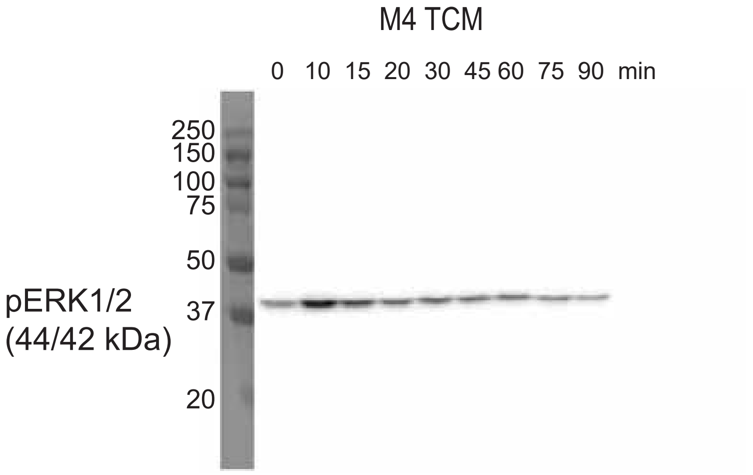

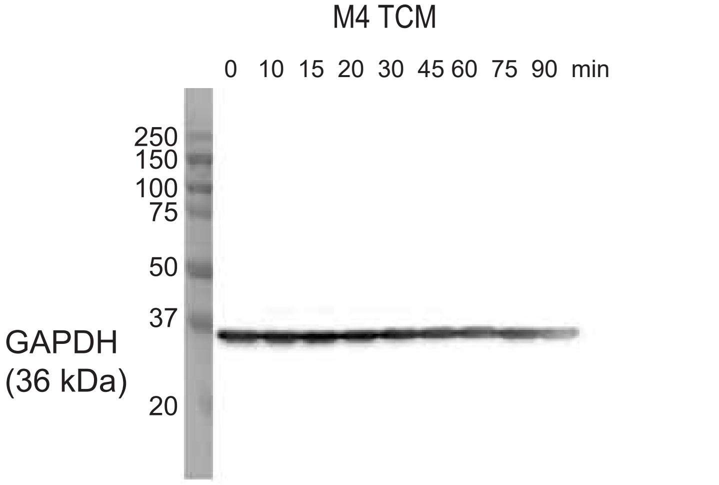

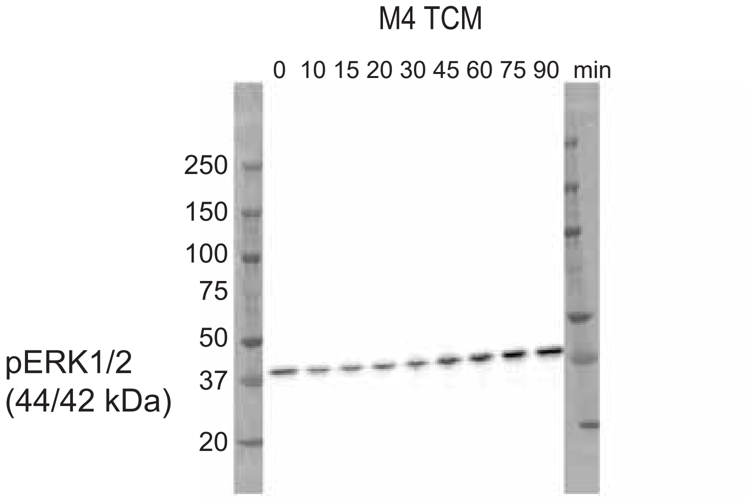

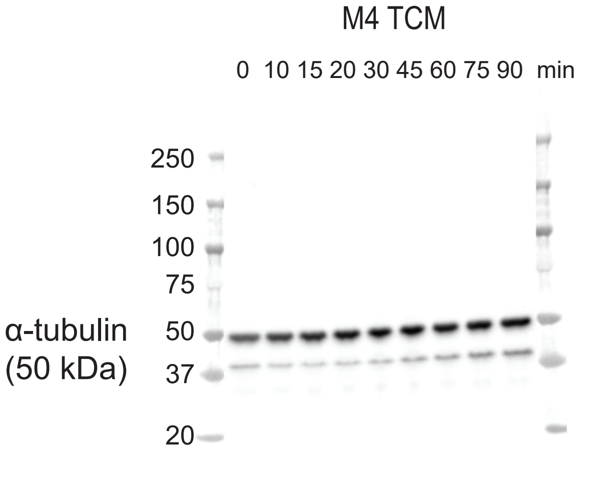

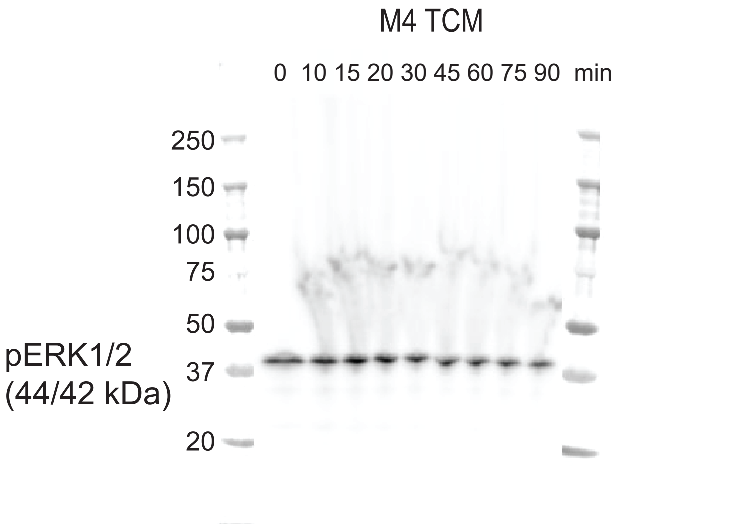

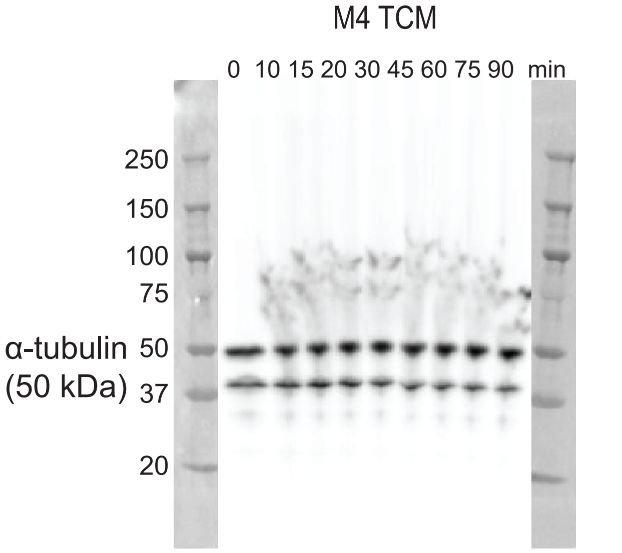

Residual pERK1/2 is seen on these loading controls.

Figure S1A-B

dHL-60 cells

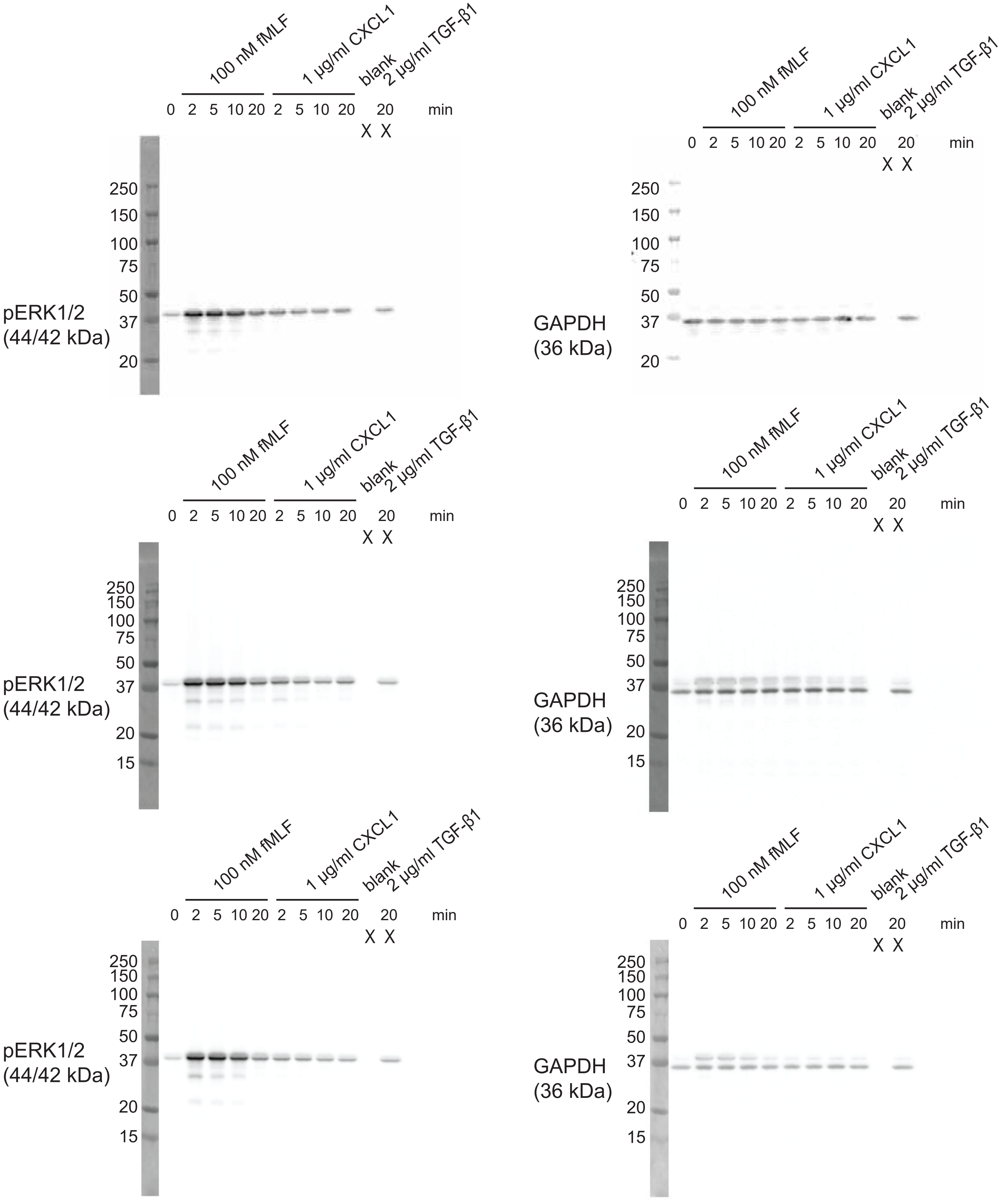

Residual pERK1/2 is seen on these loading controls.

X: Lane is not included in the final image or analysis.
