# Supplementary material for: TGF-β1 activates neutrophil signaling and gene expression but not migration
Source: PLoS One. 2023 Sep 8;18(9):e0290886. doi: 10.1371/journal.pone.0290886 (PMC10490904; doi:10.1371/journal.pone.0290886)
Supplement: Attachment — Submitted filename: Comments.docx [file pone.0290886.s007.docx]

Manuscript by Lauren E. Hein et al describes impact of TGF-β1 on neutrophil signaling, migration, and gene expression. The results convincingly show direct effects of TGF-β on neutrophils signaling and migration in vitro. This manuscript describes a detailed study, the data are clear, the experiments are well done, the paper was very clearly written, figures are comprehensive with error bars, experiments are properly deigned with controls. In sum, this is a high-quality study reporting a fascinating result, which I strongly recommend publishing after addressing minor comments. The authors not only clear the longlisting question whether TGF-β induce neutrophil chemotaxis but also made several significant new findings. The work certainly adds new knowledge to this field, and in my opinion will be of high impact. Thus, I only have a few minor comments.

Line 72: CXCR2? Detail not explained before this.

Line 282: What concentration of TGF-β1 was used for the time course, for both dHL-60 and PMNs, no mention in the figure or the figure legend?

Line 299: What is NRAS, reference 51 and 66 is about it, need some information about it in this paper too.

Figure 2, 3, and 4: What number 52, 36, 44/42, and 50 are? Size of the protein bands in blots in kDa?

Line 315: What is CXCL1? Only in LTB4 ELISA assay experimental section mentioned as a stimuli.

Line325: secretion of LTB4

Line 325 or Figure 5Ai: What concentration of fMLF was used? Is it same like figure 5B, that is 20 nM? PMNs produced on average 750 pg/ml LTB4 in response to the positive control fMLF, in Figure 5Ai it is more than 1000 pg/ml, so higher than 20 nM used?

Line338-39: What concentration of MK886, SB431542, and AZD5069 was used?

Figure 5B: When TGF-β + CXCL1 used, is the concentration same, that is 500 pg/ml for TGF-β and 100 ng/ml for CXCL1? Same for TGF-β+ IL8?

Figure 5C: SB is the abbreviation for SB431542, in previous figures full name was used and not the abbreviation, pick one and be consistent.

Line 359: What concentration of TGF-β1 was used to see the fold change expression of PAI1 and KLF10 targets (in Figure 6A)?

Line 366 and Figure S2A: In text it is IMDM and in figure it is MDM.

Figure 6F: What about CISH expression in response to M4 TCM, upregulated or downregulated, pointed out in figure like VEGFA, OSM and RGS2, but no mention in the text.

Line762 and 763: log2 fold or Log2 Fold, pick one and be consistent.
