# Supplementary material for: TGF-β1 activates neutrophil signaling and gene expression but not migration
Source: PLoS One. 2023 Sep 8;18(9):e0290886. doi: 10.1371/journal.pone.0290886 (PMC10490904; doi:10.1371/journal.pone.0290886)
Supplement: Attachment — Submitted filename: Response To Reviewers.docx [file pone.0290886.s008.docx]

**MS ID#:** PONE-D-23-16254

TGF-β1 activates neutrophil signaling and gene expression but not migration

Hein, SenGupta, Gunasekaran, Johnson, and Parent

We would like to thank the reviewers for their thoughtful feedback on the manuscript. In response to the raised comments, we have made changes to the manuscript, which has improved it.

**RESPONSE TO THE REVIEWERS COMMENTS**

**Reviewer #1**

*The research work presented aims to explore the role of TGF-β signaling in human neutrophil and neutrophil like cell line HL-60, in-vitro. The experiments are neatly designed and executed. Results interpretation and discussion is precise and easy to understand.
My recommendation: The article can be accepted in current form with few minor corrections mentioned below.*

*Line 72: CXCR2 has been used as abbreviation without any prior explanation/full form.*

The full form of CXCR2 has been added (see line 72).

*Line 299: NRAS mutation; what does NRAS stand for?*

The full form of NRAS has been added (see line 329).

*Line 315: CXCL1 has no prior explanation.*

We added that CXCL1 is a chemokine in the introduction (see line 75).

*Fig 1 B V & VI the numbers should be 2.0 and 5.0 ng/ml respectively instead of 2 and 5.*

Done. We also added the tens decimal place to Figures 1A, 1C, 2A, 2B, 2G, and 2H.

*Fig 6a: Legend has no mention of p-values. So, if the fold change is non-significant, how can one base the discussion of a non-significant result?*

We have added the statistics to Figure 6A and updated the figure legend. Using an	unpaired t-test, the fold changes for both PAI1 and KLF10 are significant.

*Protein markers have no unit mentioned in any western blot.*

Protein markers have all been updated to include ‘kDa’. This change affects Figures 2, 3,	4, and S1.

*It is great to see the authors acknowledging the effects of different media on the genes’ expression. I wonder if authors would like to make a comment or open a discussion in the scientific community about the effect of media on a system under study? Especially when the effects of media could change the expression of 100s of genes such as in this study.*

We thank the reviewer for suggesting this extension of our work. Although we agree that	it is important to evaluate potential effects of media on cells, we think that opening this	discussion to the broader topic of all cell lines and extending the discussion beyond	neutrophils is out of the scope of this manuscript.

**Reviewer #2**

*Manuscript by Lauren E. Hein et al describes impact of TGF-β1 on neutrophil signaling, migration, and gene expression. The results convincingly show direct effects of TGF-β on neutrophils signaling and migration in vitro. This manuscript describes a detailed study, the data are clear, the experiments are well done, the paper was very clearly written, figures are comprehensive with error bars, experiments are properly deigned with controls. In sum, this is a high-quality study reporting a fascinating result, which I strongly recommend publishing after addressing minor comments. The authors not only clear the longlisting question whether TGF-β induce neutrophil chemotaxis but also made several significant new findings. The work certainly adds new knowledge to this field, and in my opinion will be of high impact. Thus, I only have a few minor comments.*

*Line 72: CXCR2? Detail not explained before this.*

The full form of CXCR2 has been added (see line 72).

*Line 282: What concentration of TGF-β1 was used for the time course, for both dHL-60 and PMNs, no mention in the figure or the figure legend?*

2 ng/ml of TGF-β1 was used. This information has been added to Figures 2 and 3 as	well as the figure legends.

*Line 299: What is NRAS, reference 51 and 66 is about it, need some information about it in this paper too.*

The full form of NRAS has been added to Line 320. Reference 66 in the discussion refers to a reported role for mutated NRAS to activate the MEK/ERK pathway in melanocytes.

*Figure 2, 3, and 4: What number 52, 36, 44/42, and 50 are? Size of the protein bands in blots in kDa?*

The unit for the size of the protein bands (kDa) has been added to Figures 2, 3, 4, and S1.

*Line 315: What is CXCL1? Only in LTB4 ELISA assay experimental section mentioned as a stimuli.*

We added that CXCL1 is a chemokine in the introduction (see line 75).

*Line325: secretion of LTB4*

Done.

*Line 325 or Figure 5Ai: What concentration of fMLF was used? Is it same like figure 5B, that is 20 nM? PMNs produced on average 750 pg/ml LTB4 in response to the positive control fMLF, in Figure 5Ai it is more than 1000 pg/ml, so higher than 20 nM used?*

20 nM fMLF was used for both panels. This information has been added to Figure 5Ai.	The LTB_4_ secretion values presented in panels 5Ai and 5B are comparable, considering	the variations that occur between blood donors.

*Line338-39: What concentration of MK886, SB431542, and AZD5069 was used?*

Concentrations used were 1 µM AZD5069, 500 nM SB431542, and 100 nM MK886. This	information has been added to the legend for Figure 5.

*Figure 5B: When TGF-β + CXCL1 used, is the concentration same, that is 500 pg/ml for TGF-β and 100 ng/ml for CXCL1? Same for TGF-β + IL8 combination?*

Yes, the same concentration is used whether alone or in combination. This information	has been added to the legend for Figure 5.

*Figure 5C: SB is the abbreviation for SB431542, in previous figures full name was used and not the abbreviation, pick one and be consistent.*

The full name SB431542 has been added to Figure 5C. The full name for AZD5069 was	also included.

*Line 359: What concentration of TGF-β1 was used to see the fold change expression of PAI1 and KLF10 targets (in Figure 6A)?*

2 ng/ml TGF-β1 was used. This information has been added to the legend for Figure 6.

*Line 366 and Figure S2A: In text it is IMDM and in figure it is MDM.*

On our end, it appears that the figure shows IMDM. It is possible that it got cut off in the	margin at some point between exporting, uploading, and downloading files, so we will	check to make sure that it is shown in full before publishing.

*Figure 6F: What about CISH expression in response to M4 TCM, upregulated or downregulated, pointed out in figure like VEGFA, OSM and RGS2, but no mention in the text.*

CISH is now mentioned in the results section (see line 474).

*Line762 and 763: log2 fold or Log2 Fold, pick one and be consistent.*

Done.
